# Melatonin decreases M1 polarization via attenuating mitochondrial oxidative damage depending on UCP2 pathway in prorenin-treated microglia

**DOI:** 10.1371/journal.pone.0212138

**Published:** 2019-02-11

**Authors:** Li Hu, Shutian Zhang, Haoyu Wen, Tianfeng Liu, Jian Cai, Dongshu Du, Danian Zhu, Fuxue Chen, Chunmei Xia

**Affiliations:** 1 Laboratory of Neuropharmacology and Neurotoxicology, Shanghai Key Laboratory of Bio-Energy Crops, College of Life Science, Shanghai University, Shanghai, P.R. China; 2 Department of Physiology and Pathophysiology, Basic Medicine College, Fudan University, Shanghai, P.R. China; 3 Department of neurology, Renji Hospital, Shanghai Jiaotong University, Shanghai, P.R. China; Max Delbruck Centrum fur Molekulare Medizin Berlin Buch, GERMANY

## Abstract

Accumulating evidence suggests that neuroinflammation and oxidative stress in cardiovascular center contribute to the pathological processes underlying hypertension. Microglia activation triggers the inflammation and oxidative stress. Melatonin is a documented potent anti-inflammatory regent and antioxidant, the underlying roles of melatonin in regulating microglia activation via mitochondria remain unclear. In present study, we investigated the protective role of melatonin in decreasing M1 phenotype switching via attenuating mitochondrial oxidative damage in dependence on uncoupling protein 2 (UCP2) pathway in microglia. Prorenin (20 nmol/L; 24 hr) was used to induce inflammation in cultured microglia. Mitochondrial morphology was detected by transmission electron microscope. The reactive oxygen species (ROS) production by using DCFH-DA fluorescence imaging and mitochondrial membrane potential (MMP, ΔΨm) was evaluated by JC-1 staining. The indicator of the redox status as the ratio of the amount of total NADP^+^ to total NADPH, and the expression of 6 subunits of NADPH oxidase is measured. The pro-inflammatory cytokines releasing was measured by qPCR. UCP2 and activated AMPKα (p-AMPKα) expression were examined by immunoblot. Melatonin (100 μM) markedly alleviated the M1 microglia phenotype shifting and abnormal mitochondria morphology. Melatonin attenuated prorenin-induced ΔΨm increasing and ROS overproduction. Melatonin decreased the redox ratio (NADP^+^/NADPH) and the p47phox and gp91phox subunits of NADPH oxidase expression in prorenin-treated microglia. These effects were reversed in the presence of UCP2 siRNA. Our results suggested that the protective effect of melatonin against prorenin-induced M1 phenotype switching via attenuating mitochondrial oxidative damage depending on UCP2 upregulation in prorenin-treated microglia.

## Introduction

Accumulating evidence suggests that neuroinflammation [1,2,3] and oxidative stress [4] in cardiovascular center contribute to the pathological processes underlying sustained high blood pressure, and microglia activation have been proposed to play an important role in the progression of neuroinflammation and oxidative stress. Our previous study indicated that the rodent models which display neurogenic hypertension (e.g., stress-induced hypertension, SIH) in the brain exhibit microglia activation and increased levels of pro-inflammatory cytokines in the rostral ventrolateral medulla (RVLM), an area crucial for regulation of sympathetic outflow[5].

On activation, microglia can acquire either a neurotoxic pro-inflammatory M1 or the neuroprotective anti-inflammatory M2[6]. It was reported that a switch from a neuroprotective (M2) to a pro-inflammatory (M1) dominant response occurred in microglia during development of SIH in the hypothalamic paraventricular nucleus (PVN) of rats[7]. Unfortunately, the triggers of M1 switching remain unclear.

The regulation of microglia function via mitochondrial homeostasis is important in the development of neuroinflammation and oxidative stress damage[8]. Uncoupling protein 2 (UCP2) is the essential regulator of mitochondrial membrane potential, it is a solute carrier protein in the inner mitochondrial membrane that regulates proton leak and consequently the production of mitochondrial ROS[9,10]. UCP2 is expressed in microglia and dynamically modulates the production of ROS in response to various stimuli, and adopt microglia to M1 or M2 phenotypes, which suggesting that it may control microglial activation[11].

As the antioxidant and mitochondrial protector, melatonin (N-acetyl-5-methoxytryptamine) and its metabolites directly scavenge a variety of free radicals[12], it has been shown to inhibit microglia activation, and reduce pro-inflammatory cytokines in many experimental models including hypoxic brain injury in neonatal rats[13,14,15]. Both in vitro and in vivo studies have shown mitochondria is very sensitive to the regulatory effects of melatonin[16,17]. However, the mechanism through which melatonin acts on microglia activation is dubious. Previous literature in diabetes obesity model indicated that melatonin might regulate uncoupling proteins[18]. Nevertheless, few studies have been conducted to investigate whether melatonin could influence the uncoupling biological process.

Stimulation of microglia by lipopolysaccharide (LPS) or ROS can induce an M1 state characterized by phagocytic activity, the secretion of pro-inflammatory cytokines IL-1β, nitric oxide, TNF-α, and the generation of ROS[19,20]. Except above-mentioned factors, Peng Shi et al[21]. indicated that the renin-angiotensin-system (RAS) component prorenin elicits direct activation of hypothalamic microglia in culture and induction of pro-inflammatory mechanisms in microglia, which involve prorenin receptor-induced NF-κB activation. Thereafter, the oxidative stress responses effects of prorenin in microglia need to be further investigated. In our present study, we hypothesize that the protective role of melatonin in decreasing M1 phenotype switching depends on uncoupling protein 2 (UCP2) pathway in prorenin-treated microglia. We first examined the protective effects of melatonin against prorenin-induced M1 activation in rat primary cultured microglia. Then, the oxidative stress parameters (ROS production), the indicator of the redox status as the ratio of the amount of total NADP^+^ to total NADPH, and the expression of 6 subunits of NADPH oxidase was measured. Mitochondrial morphology, function and inflammation-related factors were evaluated. Furthermore, the UCP2 signaling pathways, which were activated by melatonin, were investigated. We proved that melatonin protected mitochondrial against oxidative stress damage and attenuated M1 switching via UCP2 pathway in prorenin-treated microglia.

## Materials and methods

### Drugs and reagents

Melatonin, dimethyl sulfoxide (DMSO), 4′,6-diamidino-2-phenylindole (DAPI), anti-β-actin (A1978) were purchased from Sigma-Aldrich (Sigma, St Louis, MO, USA). Recombinant human prorenin (ab93266) and anti-gp91phox (ab80508) was purchased from abcam (Abcam, Cambridge, MA, USA); The following antibodies were purchased from cell signaling technology (CST, Beverly, MA, USA): anti-p47phox(# 4312); anti-p-AMPKα (#2535), anti-AMPKα(# 5831), anti-NRF1(#46743), anti-TFAM (#8076), anti-UCP2(#89326), anti-CD206 (#91992) anti-CD86 (#91882) as primary antibodies; Goat anti-rabbit, goat anti-mouse secondary antibodies (Zhongshan Company, Beijing, China) were used. The mitochondria membrane potential assay kit with JC-1, ROS fluorescent probe-DCFH-DA Kit was purchased from the Beyotime Institute of Biotechnology (Nanjing, Jiangsu, China). The rat UCP2 siRNA and control siRNA was designed and purchased from HanBio Technology (Shanghai, China).

### Primary culture of rat microglial cells and experimental design in vitro

Rat primary microglia cultures were prepared from mixed glial cultures which obtained from medulla oblongata covering RVLM of 1-2-day-old newborn Sprague-Dawley rats. All experimental procedures conformed to both Institutional Animal Care and Use Committee (IACUC) of Fudan University and international guidelines on the ethical use of animals; all efforts were made to minimize the number of animals used and their suffering. Briefly, Primary cultures of microglial cells were prepared as previously described[22]. After decapitation, Medulla oblongata covering RVLM was collected and cleared of meninges, and mechanically dissected. The medulla oblongata was performed as we described previously[5]. The brain was rapidly removed and immediately frozen on dry ice. Medulla oblongata covering RVLM was blocked between 0.5 and 1.5 rostral to the obex, which was adopted from the atlas of Watson and Paxinos[23], and served as the anatomical landmark. These coordinates were selected to cover the extent of ventrolateral medulla in which functionally identified sympathetic premotor neurons reside. Both sides of the ventrolateral medulla covering RVLM (approximately at 1.5- to 2.5-mm lateral to the midline and medial to the spinal trigeminal tract) were collected by micropunches with a 1-mm inner diameter burr. Next, the minced tissue was incubated in Hank’s balanced salt solution (HBSS) dissecting medium with glucose, bovine serum albumin (BSA) and HEPES, as well as 0.025% trypsin at 37°C for 20 min. A completely dissociated suspension of the medulla oblongata was prepared by mild trituration with a fire-polished Pasteur pipette. Then, cells were plated at a density of 3 × 10^5^ cells/cm^2^ in culture medium containing of Dulbecco’s modified Eagle medium (DMEM) with GlutaMax and high glucose (4.5 g/L), supplemented with 10% fetal bovine serum (FBS), 0.1 mg/mL streptomycin with 100 U/mL penicillin on poly-l-lysine-coated 75 cm^2^ culture flasks. The culture medium was removed and replaced with fresh medium after 3 days and kept in 37°C, 95% O_2_/5% CO_2_ environment in vitro_._ On the 9^th^ day, cells were resuspended in culture medium after centrifugation (150× g for 10 min). The cells were plated at a final density of 1.2 × 10^6^ cells/well in 6-well plates and/or 2 × 10^5^ cells/well in 24-well plates[22]. As routine, trypan blue exclusion was used to determine cell viability. The cells were used for experiments 2 days after plating. The purity of cultured microglia is more than 95% evaluated by an anti-OX42 antibody of microglia marker.

For the initial in vitro experiments, microglia were assigned to 4 groups: (i) Control group; (ii) Prorenin (PRO, 20 nmol/L) treatment group for 24hr (PRO); (iii) PRO +melatonin (MEL, 100 μM) cotreatment group;(iv) PRO+ MEL +UCP2 siRNA. Melatonin stock solutions were prepared in DMSO and diluted in culture media immediately prior to an experiment. (0.1%) volume of DMSO in FBS free DMEM solution used as the control as previous described[24]. The concentration and time of incubation of SD rat primary microglia with human recombinant prorenin (20 nmol/L; 24 hr) followed Shi P et al.[21] has reported.

### RNA interference

Small interfering RNA (siRNA) targeted at rat UCP2 was used to silence UCP2. Primary microglia were cultured and incubated at 37°C in a 5% CO_2_ incubator until 70–80% confluent. Microglia were then seeded out in a 6-well plate at a density of 1.2 × 10^6^ cells/well. UCP2 siRNA duplex (10 μM) were diluted in Opti-MEM medium (Thermo Scientific). Lipofectamine RNAiMAX transfection reagent (Thermo Scientific) was also diluted in Opti-MEM medium. Then, the diluted siRNA was put to diluted lipofectamine RNAiMAX reagent (1:1 ratio). The siRNA-lipid complex was then incubated at room temperature for 5 min. The complex was thereafter added to cells and incubated for a further 24 hr. Following transfection, media was changed in transfected cells to complete media and incubated for a further 18 hr. Effects of melatonin on redox ratio, mitochondrial potential and ROS production in prorenin-stimulated control siRNA and UCP2 siRNA-transfected cells were then determined.

### Flow cytometric analysis of M1 phenotype

Microglia were harvested by washing in Dulbecco's phosphate buffered saline (PBS, without Ca^2+^ and Mg^2+^) for 20 minutes at 37°C and then resuspended in cold PBS containing 0.5% BSA/0.05% NaN_3_ to metabolically fix the cells. Microglia were stained with anti-CD86 or appropriate PE-conjugated isotype control antibodies as per the manufacturer's instructions and microglia surface staining was assessed by flow cytometry (FACS Calibur running CellQuest Pro; Becton Dickinson, UK) and analyzed using Flowing software v2.5.1.

### Analysis intracellular free radical species production by DCFH-DA fluorescent imaging

Total intracellular ROS was determined by staining microglia with dichlorofluorescin diacetate (DCFH-DA). DCFH-DA is a ROS-specific fluorescent probe, was used to measure total intracellular ROS levels. Microglia (5 × 10^5^ cells) were seeded in a 6-well plate overnight, treated as the previously described, and collected into 5-mL tubes. Cells were incubated with 10 μM DCFH-DA dissolved in DMEM without FBS at 37°C in a dark room. After washing with serum-free medium three times, the cells were analyzed by a laser scanning confocal microscope (Olympus FV 1200). To detect the mean fluorescence intensity (MFI) with an excitation wavelength of 488 nm and an emission wavelength of 525 nm.

### Mitochondrial membrane potential (MMP, ΔΨm) detection

ΔΨm of microglia (2 × 10^4^) was measured with the cationic probe: 5,5′,6,6′-Tetrachloro-1,1′,3,3′-tetraethyl-imidacarbocyanine iodide (JC-1). Red fluorescence was attributable to potential-dependent dye aggregation in the mitochondria. Green fluorescence, reflecting the monomeric form of JC-1, appeared in the cytosol following mitochondrial membrane depolarization. ΔΨm increases linearly corresponds to the formation of JC-1 aggregates and their fluorescence. Briefly, microglia were washed twice with PBS, and then loaded with JC-1 at 37°C for 20 min. After rinsing twice with the staining buffer, images were obtained using a laser scanning confocal microscope (Olympus FV 1200). The green fluorescence of JC-1 monomer was excited with a 488-nm line of helium–neon laser line and imaged through a 525-nm-long path filter. Meanwhile, the red fluorescence of JC-1 aggregation was excited with a 543-nm line of helium–neon laser line and imaged through a 590-nm-long path filter. Temperature was maintained at 37°C.

### RNA isolation and qPCR analysis

Total RNA was isolated from microglia using TRIzol reagent (Invitrogen; Thermo Fisher Scientific, Inc.) according to the manufacturer’s instructions. cDNA was transcribed from 1 mg of total RNA using Reverse Transcription System (Promega, Madison, WI). SYBR-Green-based real time quantitative PCR was performed using the iCycler (Bio-Rad Laboratories Inc, Hercules, CA). Quantification of gene expression was performed using an ABI PRISM 7500 Sequence Detection system (Applied Biosystems Life Technologies, Foster City, CA) with SYBR Green (TransGen Biotech Co., Ltd.). Sense and antisense primer sequences were in Table 1.

**Table 1:** Sense and antisense primer sequences.

Gene	Primer sequence	
	Forward	Reverse
IL-1β	5’-AATCTCACAGCAGCACATCAA-3’	5’-AGCCCATACTTTAGGAAGACA-3’
TNF-α	5’-CCCCTCAGCAAACCACCAAGT-3’	5’-CTTGGGCAGATTGACCTCAGC-3’
p40phox	5’-AGAGCGACTTTGAGCAGCTT-3’	5’-TGTGGAGACACACCCTTGAT-3’
p47phox	5′-GGTGGGTCATCAGGAAAGAC-3′	5′-GCAGAAAACGGACGCTGTTG-3′
p67phox	5′-GCCAGGTGAAAAACTACTGC-3′	5′-CTTCCAGCCATTCTTCATTC-3′
Rac1	5′-ATGCAGGCCATCAAGTATGTGGTG-3′	5′-TTACAACAGCAGGCATTTTCTCTTCC-3′
gp91phox	5′-CTTGGATGATAGCACTGCAC-3′	5′-CTTCATCTGAAGCTCAATGG-3′
p22phox	5′-TGTGCCTGCTGGAGTACCCC-3′	5′-ACACGACCTCGTCGGTCACC-3′
β-actin	5’-CCACACCCGCCACCAGTTCG-3’	5’-CCCATTCCCACCATCACACC-3’

The experiments were repeated three times with triplicates. The results were expressed as fold changes between the sample and the controls corrected with internal control.

### Western blot

Microglia were collected in lysis buffer (1% SDS, 50mM Tris pH 7.4, 1mM EDTA, 10 μl/ml protease inhibitors and 10 μl/ml phosphatase inhibitors) and protein content was determined by using the Lowry protein assay. Concentrated conditioned media and cell samples (15 μg total protein) resuspended with 4× Loading Buffer were loaded into 10% sodium dodecyl sulfate polyacrylamide gels (SDS-PAGE; Bio-Rad). After electrophoresis and transfer onto nitrocellulose membranes, following primary antibodies were used: rabbit anti-p-AMPKα, rabbit anti-AMPKα, rabbit anti-NRF1, rabbit anti-TFAM, rabbit anti-UCP2, mouse polyclonal anti-gp91phox, mouse polyclonal anti-p47phox (all 1:1000 dilution) and mouse anti-β-actin (1:5000). Membranes were then incubated with specific secondary antibodies conjugated to horseradish peroxidase for 90 min at room temperature in PBS-0.1% Tween-20. Labeled proteins were visualized by using the Clarity Western ECL Substrate (Bio-Rad) and detected using Bio-Rad Image Lab software with a ChemiDoc MP imaging system (Bio-Rad).

### Transmission electron microscopy

The procedures were described as previous[5]. Briefly, for electron microscopy (EM) embedding, the cell medium was decanted, and Karnovsky's fixative (2% paraformaldehyde plus 2.5% glutaraldehyde in 0.1 m phosphate buffer, pH 7.2–7.4) was added to a depth of about 5 mm. Microglia were fixed for 1–2 hr at room temperature and then overnight at 4–10°C. microglia were then osmicated, rinsed with phosphate buffer, dehydrated, and embedded in Epoxy resin, which was allowed to polymerize for 24 h at 70°C. Blocks containing microglia were sectioned using an ultramicrotome (Ultracut; Leica) at 70–80 nm. Thin sections were collected on grids and stained with uranyl acetate and lead citrate. Grids were examined under a transmission electron microscope (H-700; Hitachi, Tokyo, Japan) at 80 kV.

### Measurement of redox status and the expression of six subunits of NADPH oxidase

Because NADPH oxidase activation is closely related to the promotion of oxidant production. The reduced (NADPH) and oxidized (NADP^+^) forms of the nucleotide were measured. Intracellular NADPH and NADP^+^ production was measured using EnzyChrom NADP^+^/NADPH assay kit (BioAssay Systems ECNP-100), according to the manufacturers' protocols. microglia were treated as previously described. The relative levels were analyzed on the microplate reader (Molecular Devices Flexstation 3, USA). The expression of four cytosolic subunits (p40phox, p47phox, p67phox, and Rac1) and two membrane subunits (gp91phox and p22phox) of NADPH oxidase was evaluated.

### Statistical analysis

Statistical analyses were performed with SPSS software (version 17.0; SPSS, Inc., Chicago, IL, USA). For experiments that involved two groups of samples, Student’s unpaired t test was used. For experiments that involved multiple groups, one-way or two-way analysis of variance with repeated measures were used to assess group means. This was followed by the Tukey’s multiple range tests for post hoc assessment of individual means. Data are expressed as mean ± SEM. *P* < 0.05 was considered statistically significant.

## Results

### Prorenin-induced M1 phenotype switching and increased proinflammatory cytokines (PICs) releasing, while melatonin reversed these effects which depended on UCP2

**Fig 1:**
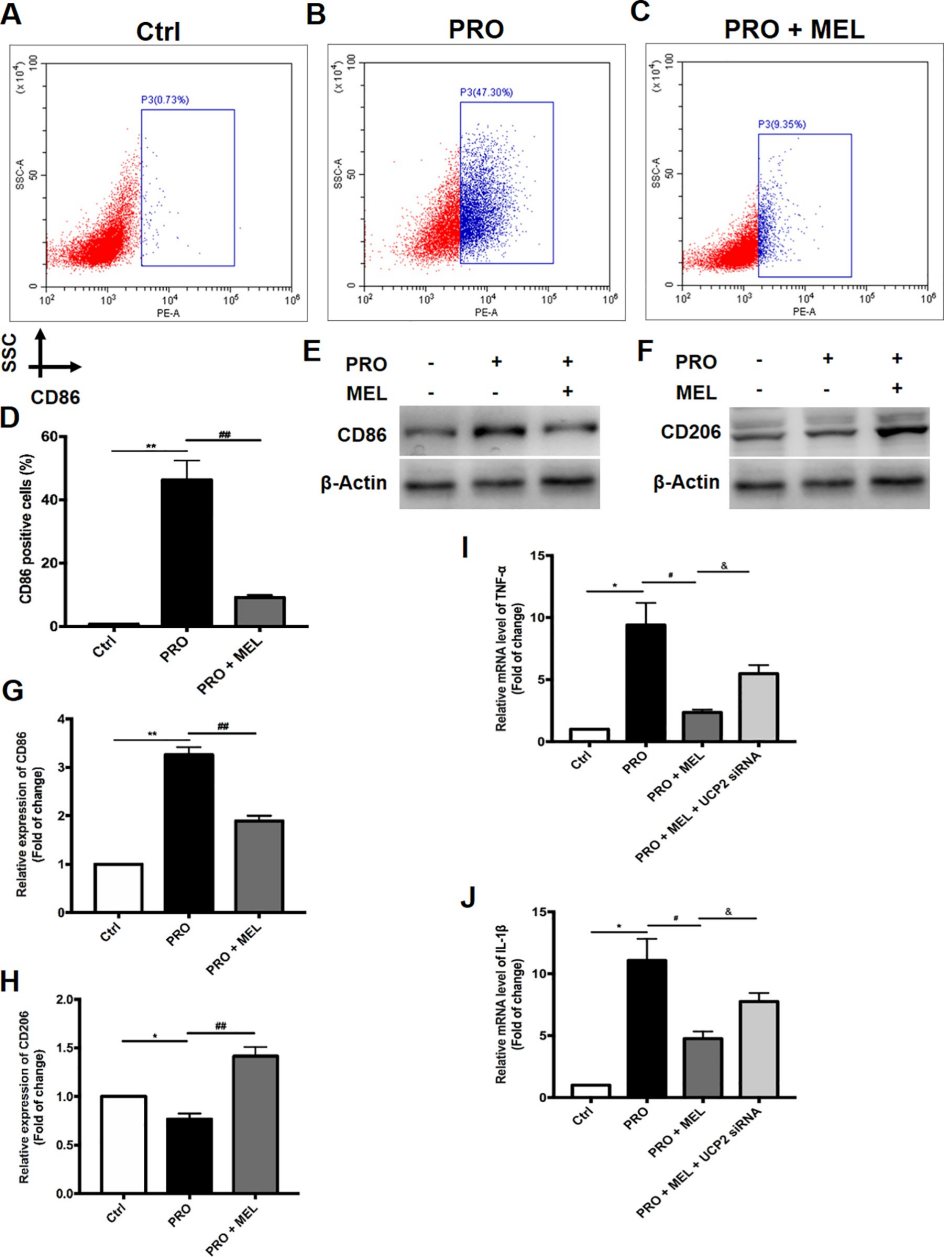
Prorenin increases M1 phenotype (CD86-positive) switching in primary microglial cells. Cells were treated with control medium or prorenin (20 nmol/L) for 24 hr, followed by analysis of M1 phenotype (CD86-positive) cells by using flow cytometry. (A–D) SSC: side scatter. Western blot analysis and representative bands of CD206 and CD86 expression were shown. Membranes were re-probed for β-actin expression to show that similar amounts of protein were loaded in each lane. (E-H) IL-1β and TNF-α mRNA levels were quantified by real time PCR. Primary microglia were treated with control solution (DMEM) or prorenin (20 nmol/L) in the presence or absence of melatonin (100 μM) and UCP2 siRNA transfection. (I-J) Statistical significance between groups was evaluated by one-factor ANOVA. Data are mean ± SEM. n = 5, *p < 0.05, **p < 0.01 vs. the control group, ^#^p < 0.05, ^##^p < 0.01 vs. PRO group, ^&^p < 0.05 vs.100 μM MEL-treated group.

Primary microglia were treated with control medium or prorenin followed by analysis of CD86-positive (a marker expressed by M1 phenotype microglia) cells by using flow cytometry. Incubation of SD rat primary microglia with recombinant human prorenin (20 nmol/L) for 24 hr elicited significant increases in the percentage (from 0.73% to 47.30%, n = 5, p = 0.032) of M1 microglia, while melatonin could reverse this effect (9.35%, n = 5, p = 0.008) (Fig 1A–1D). In addition, protein levels of M1 (CD86) and M2 markers (CD206) of microglia were tested by western blot, which showed that the protein levels of CD86 was 3.2-flod higher than that of control group (n = 5, p = 0.006), while melatonin reversed these effects (Fig 1E–1H), which implied that melatonin protected microglia from M1 phonotype shifting. To characterize inflammatory response in prorenin-stimulated microglia for 24 hr, we examined the expression of two major proinflammatory cytokines (IL-1β and TNF-α). The stimulatory effects of prorenin on IL-1β and TNF-α mRNA levels were depressed 2-fold (n = 5, p = 0.024) by co-treatment of melatonin in rat primary microglial cells (Fig 1I and 1J), which meant melatonin treatment reversed the inflammatory responses of prorenin. In addition, our results showed UCP2-silencing could eliminate the anti-inflammation effect of melatonin (n = 5, p = 0.016), which suggested that the protective effect of melatonin partially depended on UCP2. All these experiments were repeated thrice and one representative result was illustrated and shown.

### Melatonin reduced mitochondrial potential and decreased prorenin-induced reactive oxygen species (ROS) overproduction, while the effects of melatonin were abolished in the presence of UCP2 siRNA

**Fig 2:**
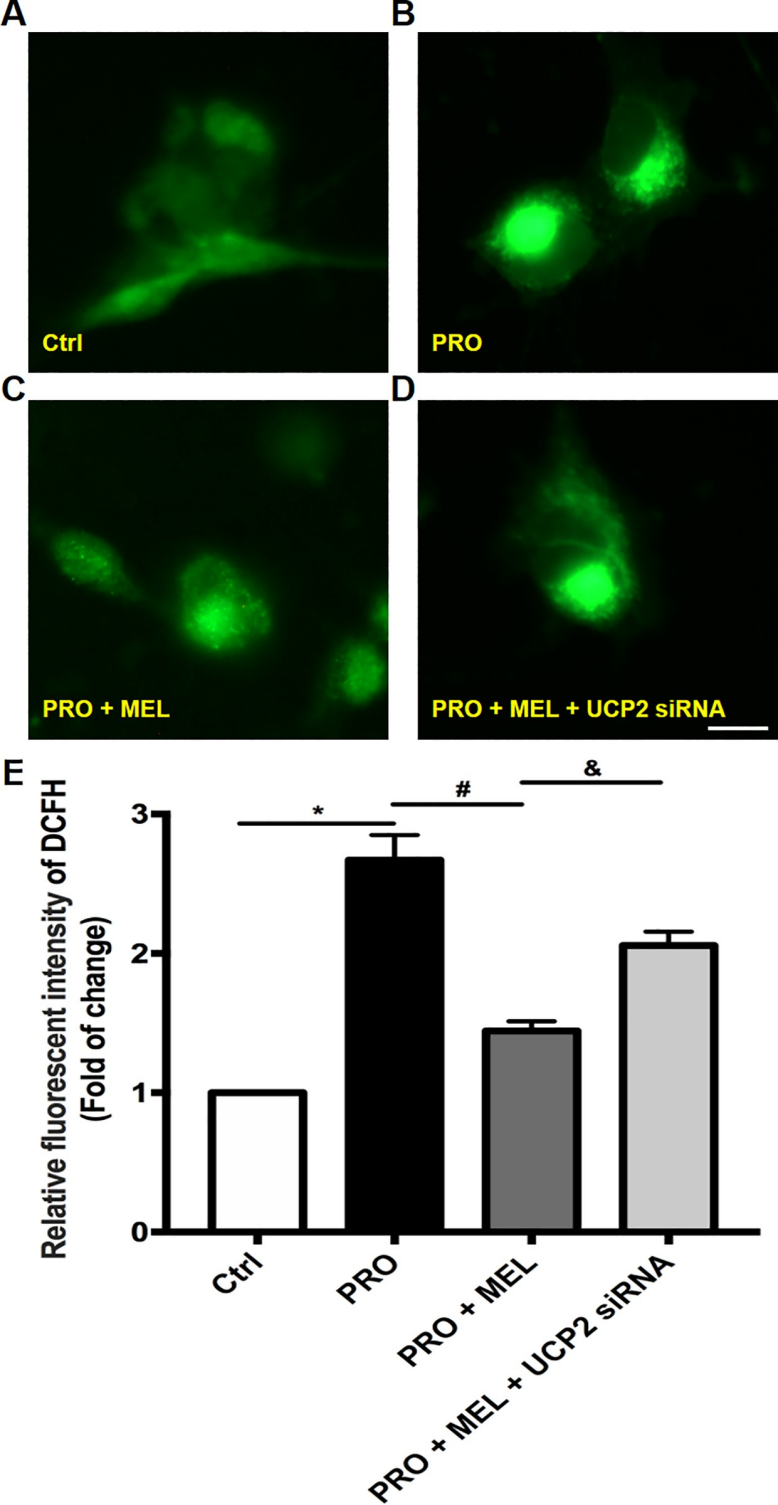
ROS production was evaluated by using DCFH-DA-associated fluorescent microscopic photographs and histogram. ROS production in control, PRO, MEL+PRO with or without UCP2 siRNA-treated in microglia (A–D) and related quantification (E) were shown. The histogram showed melatonin attenuated the ROS levels compared to that of prorenin. While in the presence of UCP2 siRNA, melatonin could not reduce the level of ROS levels. Statistical significance between groups was evaluated by one-factor ANOVA. Data are mean ± SEM. n = 3, *p < 0.05 vs. the control group, ^#^p < 0.05 vs. PRO group, ^&^ p < 0.05 vs.100 μM MEL-treated group. Scale bar: 10 μm.

ROS production was detected by DCFH-DA fluorescence readily, Primary microglia were treated with control solution (DMEM) or prorenin (20 nmol/L) in the presence of melatonin (100 μM) with or without UCP2 siRNA. Melatonin attenuated 1.5-fold of prorenin-induced ROS overproduction (n = 5, p = 0.029), while this effect was abolished in the presence of UCP2 siRNA (n = 5, p = 0.031), which meant this effect of melatonin might depend on UCP2. (Fig 2A–2E).

### Protective effect of melatonin against prorenin-induced oxidative stress was largely attributed to the decreasing of NADPH oxidase-mediated ROS production

**Fig 3:**
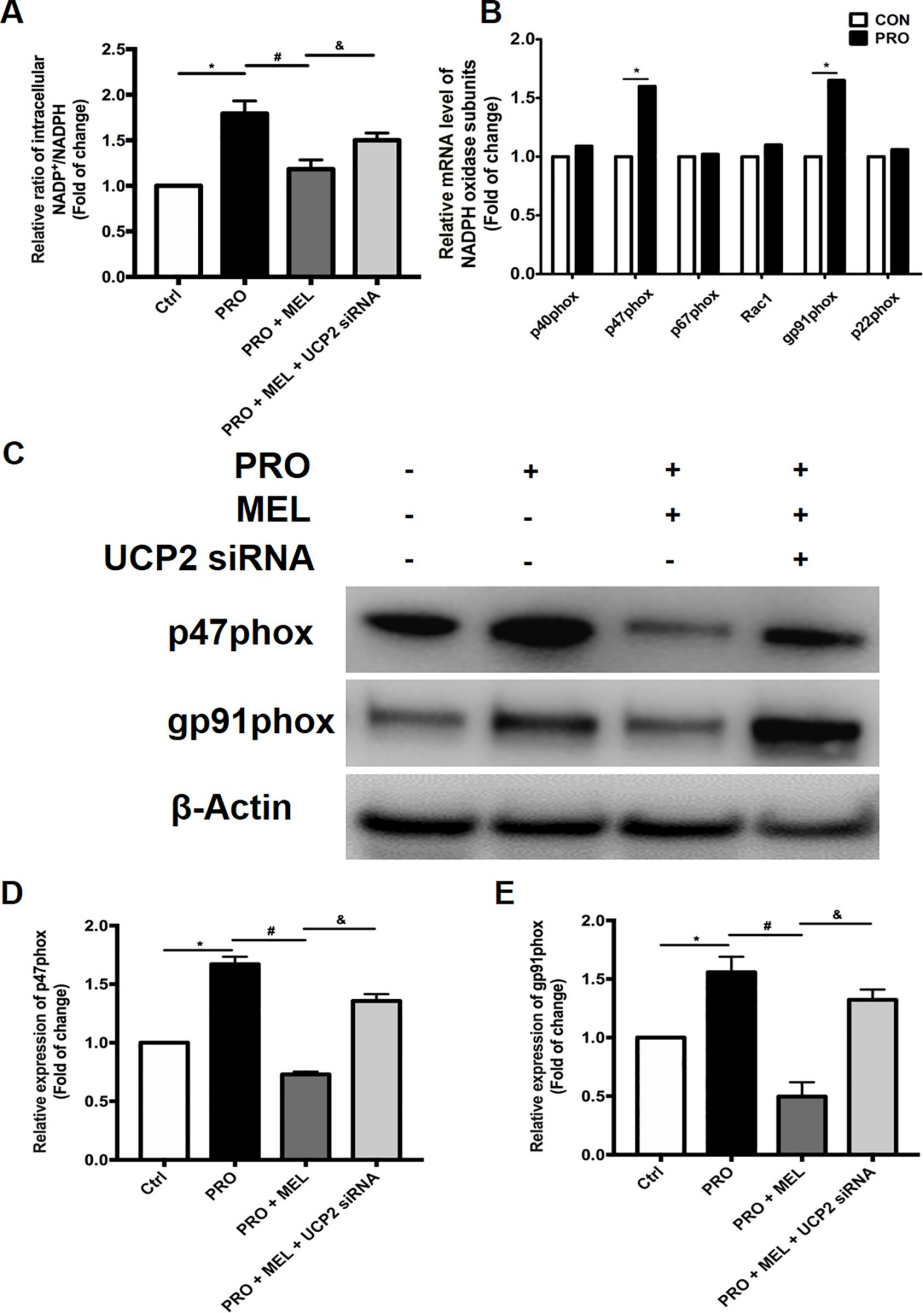
Melatonin decreased NADP^+^/NADPH Ratio and the expression of p47phox and gp91phox subunit of NADPH oxidase in prorenin-treated microglia. Prorenin increased the NADP^+^/NADPH Ratio in microglia. Melatonin considerably decreased the redox ratio (NADP^+^/NADPH) in prorenin-treated microglia. (A) The mRNA level of 6 subunits of NADPH oxidase were measured in different treated groups. (B) The expression levels of p47phox and gp91phox subunit of NADPH oxidase were markedly inhibited in the melatonin treated groups. These effects were reversed by melatonin with UCP2 siRNA presence. (C-E) Statistical significance between groups was evaluated by one-factor ANOVA. n = 3, *p < 0.05 vs. the control group, ^#^p < 0.05 vs. PRO group, ^&^ p < 0.05 vs.100 μM MEL-treated group.

As shown in (Fig 3A), the ratio of NADP^+^/NADPH were significantly increased in the prorenin group compared with those in the control group (n = 3, p = 0.043. Melatonin considerably decreased the redox ratio (NADP^+^/NADPH) (32%) in prorenin-treated microglia. While the ratio of NADP^+^/NADPH in melatonin showed significant difference in the presence of UCP2 siRNA (n = 3, p = 0.022). Then we measured the six subunits mRNA expression of NADPH oxidase: four cytosolic subunits (p40phox, p47phox, p67phox, and Rac1) and two membrane subunits (gp91phox and p22phox). And we found that the expression levels of p47phox and gp91phox subunits of NADPH oxidase were increased markedly (Fig 3B). The protein expression of p47phox and gp91phox subunits was inhibited in the melatonin-treated groups compared with those in the prorenin group (n = 3, p = 0.018) (Fig 3C–3E). From these results, we concluded that the protective effect of melatonin against prorenin-induced oxidative stress was largely attributed to the decreasing of NADPH oxidase-mediated ROS production.

### Melatonin reduced mitochondrial potential, while the effects of melatonin were abolished in the presence of UCP2 siRNA

**Fig 4:**
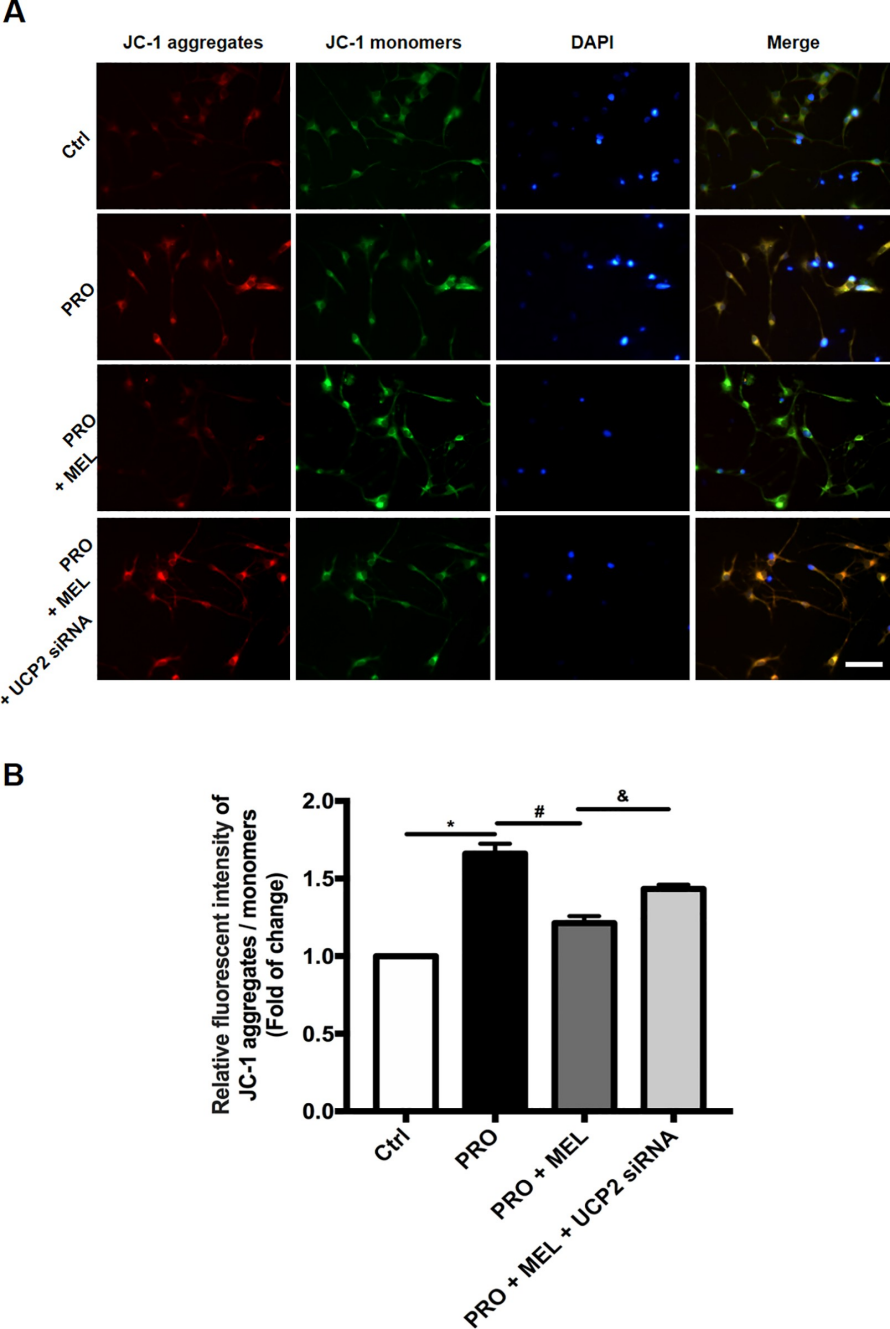
Membrane potential (ΔΨm) was measured by using a fluorescent probe JC-1 assay system in microglia. The ratio of fluorescence intensities Ex/Em = 490/590 and 490/530nm (FL590/FL530) were recorded to delineate the ΔΨm level of sample. ΔΨm of control, PRO, MEL+PRO with or without UCP2 siRNA-treated microglia (A) and related quantification (B) was shown. The average of three to five determinations for each group is shown. Statistical significance between groups was evaluated by one-factor ANOVA. Data were mean ± SEM. n = 3, *p < 0.05 vs. the control group, ^#^p < 0.05 vs. PRO group, ^&^ p < 0.05 vs.100 μM MEL-treated group. Scale bar: 30 μm.

The mitochondrial membrane potential (ΔΨm) were measured in the rat primary using a fluorescence probe JC-1 assay system. The ratio of fluorescence intensities Ex/Em = 490/590 and 490/530nm (FL590/FL530) were recorded to delineate the ΔΨm level of sample. The relative ratios of FL590/FL530 geometric mean between groups were calculated to represent the overall level of ΔΨm. As shown in (Fig 4A and 4B), the levels of the ΔΨm with an average 1.7 fold change of the mean value measured in the prorenin (n = 3, p = 0.037), which was deceased by melatonin (1.1 fold change, n = 3, p = 0.027), in contrast, the levels of ΔΨm in the presence of UCP2 siRNA was comparable with that of measured in the melatonin group (1.4-fold change vs 1.1 fold change, n = 3, p = 0.025), which implied that this effect of melatonin might depend on UCP2.

### Melatonin treatment upregulation of UCP2 and activation of AMPKα (p-AMPKα) with its downstream signaling (NRF1 and TFAM)

**Fig 5:**
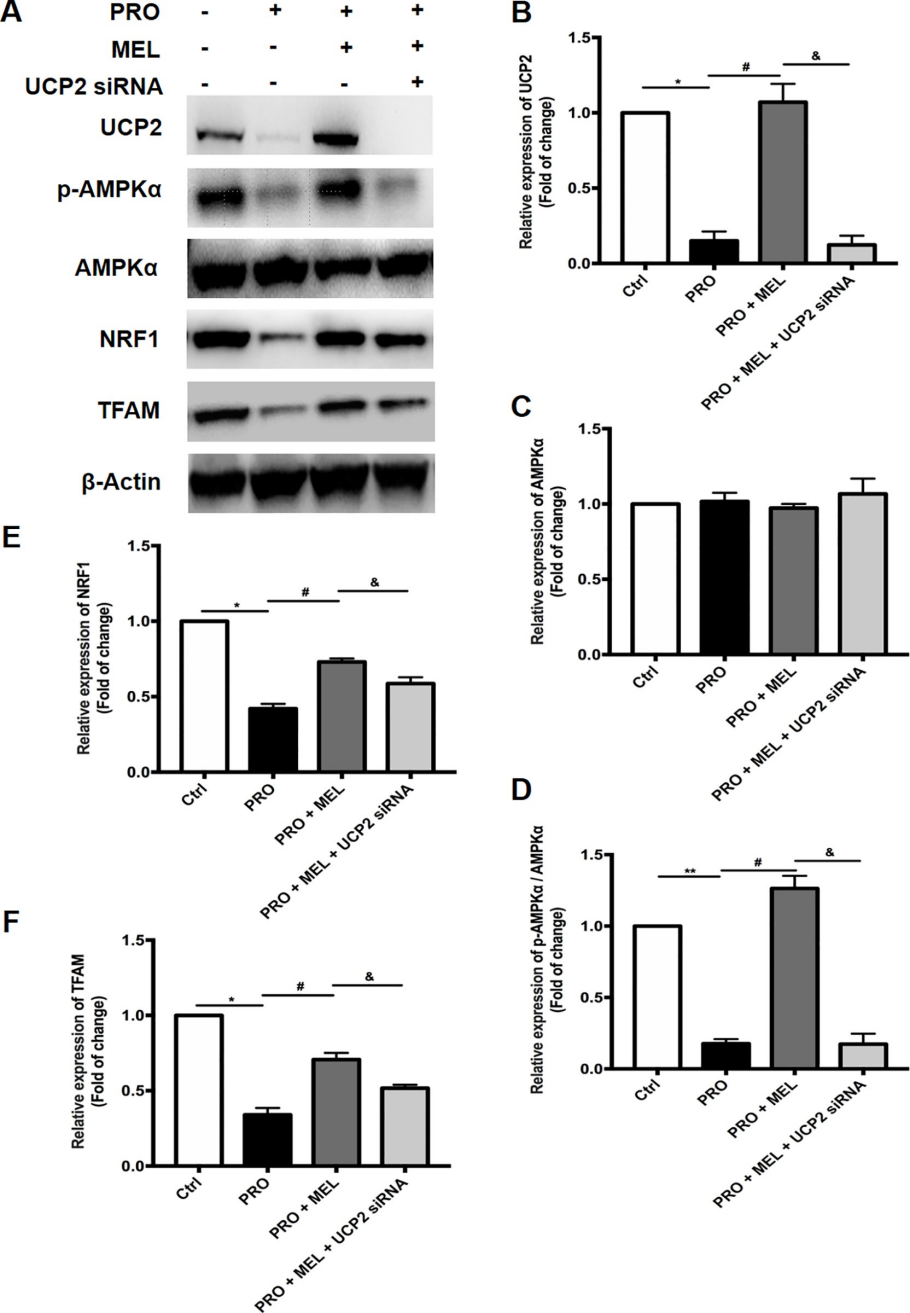
Effect of melatonin treatment combined with UCP2 siRNA on its downstream pathway. Representative immunoblot band of p-AMPKα, PGC1α, NRF1 and TFAM are shown (A), and the control group are defined as 100% in histogram (B-F). Membranes were re-probed for β-actin expression to show that similar amounts of protein were loaded in each lane. Statistical significance between groups was evaluated by one-factor ANOVA. Data are mean ± SEM. n = 3, *p < 0.05 vs. the control group, ^#^p < 0.05 vs. PRO group, ^&^p < 0.05 vs.100 μM MEL-treated group.

**Fig 6:**
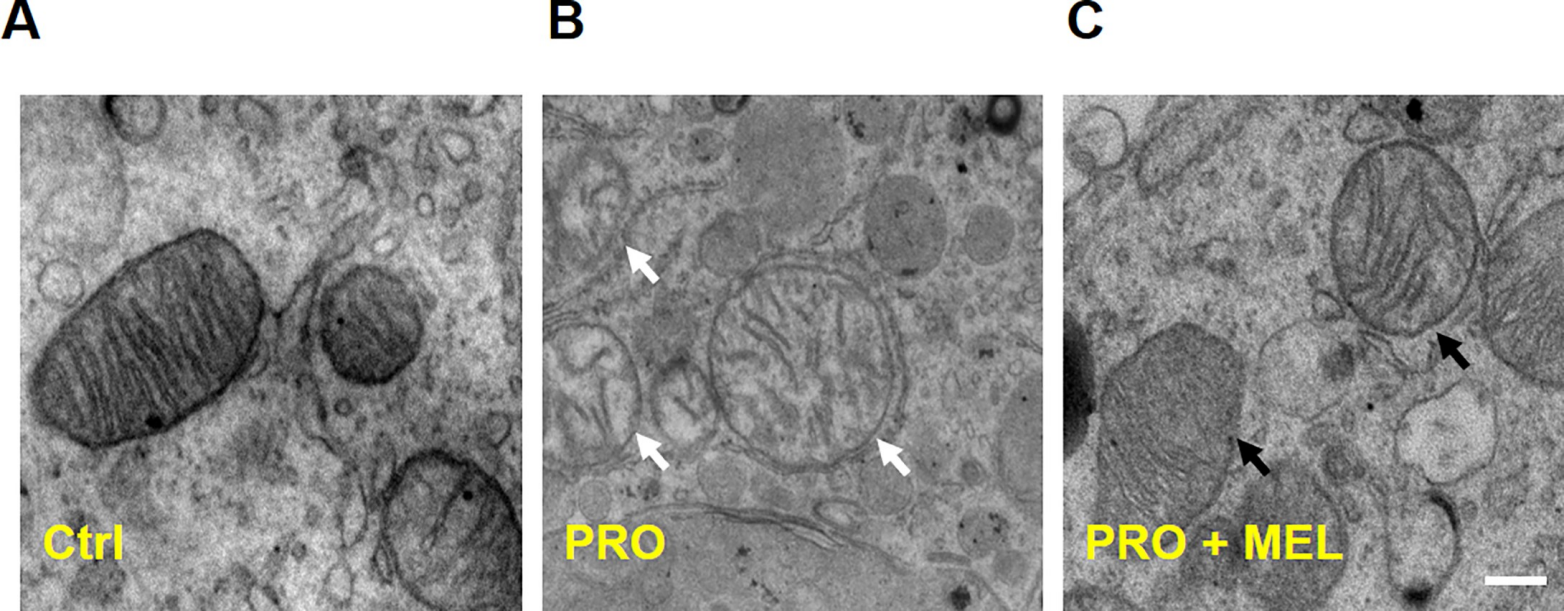
Transmission electron microscopic examination of ultrastructural morphology of mitochondria in microglia. Figures showed representative electron micrographs of microglia. The left column showed normal ultrastructural features of mitochondria (A), White arrows pointed to mitochondria with “dissolving” features in prorenin-stimulated microglia (B), and the right column showed melatonin cotreatment protected the normal ultrastructural morphology of the mitochondria (C), which revealed that melatonin markedly alleviated mitochondrial swelling, cristae disorientation and breakage compared with the prorenin-treated group. Scale bar = 1 nm.

Activation of AMPKα are responsible for maintaining mitochondrial homeostasis under various perturbations of cardiomyocytes and cells[25,26]. To further explore the underlying molecular mechanisms that melatonin exerted on mitochondria protection in microglia, the western blot analysis to be used as estimate the expression of p-AMPKα and its downstream pathway (NRF1 and TFAM). Western blot analysis revealed that prorenin-exposure for 24 h markedly inhibited the AMPKα activation (i.e., AMPKα phosphorylation) and its downstream signaling proteins (NRF1 and TFAM) expression in microglia (n = 3, p = 0.037, Fig 5A–5F). However, melatonin 100 μM cotreatment significantly upregulated the UCP2, p-AMPKα, NRF1and TFAM levels compared with PRO group (n = 3, p = 0.038, Fig 5A–5F), which suggested melatonin inhibited M1 microglia shifting via activation of UCP2 signaling pathways. Moreover, Upregulation of p-AMPKα, NRF1 and TFAM levels was further attenuated by UCP2 siRNA cotreatment with MEL (n = 3, p = 0.029, Fig 5A–5F). Further, we found enlarged mitochondria with “dissolving” features as disorder and indistinct cristae emerged in prorenin-stimulated microglia in comparison with control (Fig 6A and 6B) by using transmission electron microscope, while there is little “dissolving” mitochondrion in melatonin-treated microglia (Fig 6C). These results further demonstrated the protective effect of melatonin on mitochondria morphology.

## Discussions

The principle findings in our study were as follows. First, Prorenin-induced M1 phenotype shifting, ROS production and increased PICs (IL-1β and TNF-α) releasing. Second, melatonin reduced mitochondrial potential and decreased prorenin-induced reactive oxygen species (ROS) overproduction, and the protective effect of melatonin against prorenin-induced oxidative stress was largely attributed to decrease expression of the p47phox and gp91phox subunit of NADPH oxidase, which implied that NADPH oxidase-mediated ROS overproduction. Third, the melatonin treatment upregulation of UCP2 and activation of AMPKα (p-AMPKα) with its downstream signaling (NRF1 and TFAM), In addition, the above-mentioned effects of melatonin were abolished in the presence of UCP2 siRNA. Fourth, we found enlarged mitochondria with “dissolving” features as disorder and indistinct cristae manifested in prorenin-stimulated microglia under transmission electron microscope, while there is little “dissolving” mitochondrion in melatonin-treated microglia. Our results suggested that the protective effect of melatonin against prorenin-induced M1 phenotype switching via attenuating mitochondrial oxidative damage depending on UCP2 upregulation in prorenin-treated microglia.

Peng Shi et al.[21] indicated that prorenin, the renin-angiotensin-system (RAS) component, elicits direct activation of hypothalamic microglia in culture and induction of pro-inflammatory mechanisms in microglia, which proved that renin-angiotensin-system-induced neurogenic hypertension is not restricted to actions of angiotensin II alone. Our present study showed prorenin-induced M1 activation with increased ROS and inflammatory factors releasing, which is consistent with theirs. In addition, we found that prorenin-induced oxidative stress was largely attributed to decrease expression of the p47phox and gp91phox subunits of NADPH oxidase. NADPH oxidase is composed of four cytosolic subunits (p40phox, p47phox, p67phox, and Rac1) and two membrane subunits (gp91phox and p22phox). Active oxidase (O^2-^) generates superoxide by transferring reducing equivalents from NADPH or NADH to oxygen. Evidences have indicated that NADPH oxidase is extensively expressed in tissues and is the major source of ROS in oxidative-related hypertension animals[27,28]. Our results implied that NADPH oxidase-mediated prorenin-induced ROS overproduction in microglia.

Mitochondria contribute to the oxidative stress-related neural mechanism of hypertension. The regulation of microglia function via mitochondrial homeostasis is important in the development of neuroinflammation and oxidative stress damage[8]. Melatonin exerts receptor-dependent and receptor-independent actions involve autonomic, vascular, anti-inflammatory, and antioxidant effects. Not only melatonin, but its metabolites have antioxidant effects[29,30]. Our and others previous study have shown melatonin regulates blood pressure both by central and peripheral interventions, in addition to its relationship with the renin-angiotensin system[31,32,33,34]. In many neurodegenerative disorders, microglia are persistently activated in the M1 state, which may either be the cause or consequence of high ROS levels[35].

It has been reported ROS can trigger a phenotypic switch in both astrocytes and microglia, UCP2 activity is protective against ROS-induced cell death in the CNS [36] via reducing the inflammatory response of glia to oxidative stress. Locating on the inner mitochondrial membrane, the intrinsic of UCP2 is H^+^ channel promoting the proton leak, which can influence ΔΨm[37]. Uncoupling implies the collapse of the △Ψm caused by proton leakage from the intermembrane space to the matrix[38]. Currently, it was known that the Ca^2+^ overload can result in ROS production and mitochondrial injury, UCP2 overexpression can inhibit the depolarization of ΔΨm which can decrease Ca^2+^ uptaking into the mitochondrial matrix[39,40]. De Simone et al. demonstrated that microglial UCP2 deficiency increases the LPS-stimulated secretion of the M1 markers nitrite, TNF-α, and IL-6. More interesting, they also found a correlative relationship between levels of IL-4, which stimulates an M2 phenotype and the transcription of UCP2. They concluded that the mitochondrial UCP2 is a master regulator of both M1 and M2 microglial responses[41]. In our present research, we found that melatonin attenuated M1 phenotype switching. Effects of melatonin regulate microglial phenotypes via UCP2 denote a significant role for melatonin depending on UCP2 in the glial response to cellular stress, which imply that increases in UCP2 will be neuroprotective by reversing the production of inflammatory factors and increasing or preserving the supply of endogenous antioxidants.

It was well known that the ATP production, cellular metabolism regulation and homeostasis maintaining are closely linked to mitochondrial morphology, which is controlled by the dynamics of fission and fusion[42,43].

We found melatonin reduced enlarged mitochondria with “dissolving” features as disorder and indistinct cristae. It was reported that fission of mitochondria is associated with M1 macrophages, while fusion of mitochondria was essential for M2 macrophages[44]. Melatonin modulate mitochondrial biogenesis and dynamics by reduction of fission and elevation of fusion. In systems studied, melatonin suppresses the translocation of fission 1 protein (Fis1) and Drp1 to the outer mitochondrial membrane, thereby attenuating fission[45]. Melatonin promote mitochondrial fusion by increasing expression of mitofusins (Mfn1) via Notch1 signaling[46,47]. Our results showed that melatonin inhibited M1 microglia polarization through reducing dissolving mitochondria. Microglia involve in surveillance and are the resident immune cells of the brain, while macrophage, locates in the peripheral tissue and production of cytokines and trophic factors[48]. They share a lot of similarities, on this background, Therefore, it will be essential to further study microglia polarization in its dependence of the mitochondrial fission and fusion balance.

AMP-activated protein kinase (AMPK) is a heterotrimeric complex composed of a catalytic α subunit and regulatory β and γ subunits[49]. Phosphorylation of AMPKα in the Thr172 site significantly boosts AMPKα activity, thus maintaining the energy balance by the direct phosphorylation of target proteins which control of target genes at transcriptional level[24]. In the present study, we observed that melatonin treatment significantly activation of AMPKα in microglia and its downstream signaling (NRF1and TFAM) expression in prorenin-treated microglia. NRF1 control many genes and maintain mitochondrial function at transcriptional level, which encoding the mitochondrial enzymes, respiratory complexes, and transcription factors subunits (e.g., TFAM) that regulate mtDNA transcription and replication[50,51,52]. Our results suggested that melatonin involved maintaining mitochondrial function via regulating UCP2/AMPK signaling pathway, thereafter decreased M1 phenotype shifting. The mechanisms on interaction between melatonin and UCP2 in mitochondria of microglia need to be further investigated.

## Conclusion

Our results suggested that melatonin involved maintaining mitochondrial function via regulating UCP2/AMPK signaling pathway, which protected mitochondrial against oxidative stress damage and attenuated M1 switching in prorenin-treated microglia.

## Abbreviations

UCPs: uncoupling proteins
UCP2: uncoupling protein 2
AMPK: adenosine monophosphate activated protein kinase
NRF1: nuclear respiratory factor 1
TFAM: mitochondrial transcription factor A
ROS: reactive oxygen species
MMP, ΔΨm: mitochondrial membrane potential
RVLM: rostral ventrolateral medulla
SIH: stress-induced hypertension
RAS: renin-angiotensin-system
PVN: hypothalamic paraventricular nucleus
